# Travelling models and the challenge of pragmatic contexts and practical norms: the case of maternal health

**DOI:** 10.1186/s12961-017-0213-9

**Published:** 2017-07-12

**Authors:** Jean-Pierre Olivier de Sardan, Aïssa Diarra, Mahaman Moha

**Affiliations:** LASDEL, Niamey, Niger

## Abstract

As in other areas of international development, we are witnessing the proliferation of ‘traveling models’ developed by international experts and introduced in an almost identical format across numerous countries to improve some aspect of maternal health systems in low- and middle-income countries. These policies and protocols are based on ‘miracle mechanisms’ that have been taken out of their original context but are believed to be intrinsically effective in light of their operational devices.

In reality, standardised interventions are, in Africa and elsewhere, confronted with pragmatic implementation contexts that are always varied and specific, and which lead to drifts, distortions, dismemberments and bypasses. The partogram, focused antenatal care, the prevention of mother-to-child transmission of HIV or performance-based payment all illustrate these implementation gaps, often caused by the routine behaviour of health personnel who follow practical norms (and a professional culture) that are often distinct from official norms – as is the case with midwives.

Experiences in maternal and child health in Africa suggest that an alternative approach would be to start with the daily reality of social and practical norms instead of relying on models, and to promote innovations that emerge from within local health systems.

## Background

In the field of maternal health (as in the field of public health and even, in a more general sense, in the field of development), interventions promoted by international organisations, bi-lateral cooperation and non-governmental organisations (NGOs) in low- and middle-income countries (LMICs), and even more so in Africa, are most often highly standardised. This is usually done both with a view to simplification (given significant deficits in infrastructure and skills) and for the sake of widespread dissemination (through a search for high-impact factors). The biomedical culture, which relies heavily on statistical approaches and harmonised protocols, amplifies this trend. Consequently, there has been a growing number of ‘travelling models’ developed by international experts to improve aspects of maternal health systems in LMICs, being introduced in an almost identical format in many countries.

These standardised interventions are, in Africa as elsewhere, subjected to the formidable test of implementation contexts, which are always very diverse and largely unknown to the promoters of the interventions. The hazards and difficulties of implementing public policies have long been emphasised in the political sciences [1–5], and in development anthropology [6, 7], but they remain relatively poorly documented in public health despite a gradual increase in awareness [8–13]. Public health is dominated by the assimilation of any evidence-based medicine (and of any evidence-based policy) with data that are essentially quantitative and experimentalist (for an advocacy paper concerning maternal health see Miller et al. [14]), and which favour and justify the standardisation of interventions. In recent years, however, various theoretical currents have emphasised the importance of context in health interventions, be it realist approaches to evaluation [15, 16], approaches emphasising the complexity of health systems [17, 18], implementation studies [19, 20], or calls to take ‘real-world’ contexts into account [21, 22]. Most of these use a qualitative or a mixed methods approach. Nevertheless, the standardisation of public health interventions is still rarely contested, neither at the operational nor at the theoretical level, and as such it appears to be ‘untouchable’. Its responsibility for implementation failures is neglected, and the major role of travelling models in the ignorance or underestimation of context is largely overlooked.

Here, we develop a grounded theory [23] to better understand the relationship between standardised interventions and implementation contexts, and the many unexpected, invisible or perverse effects that result from the hegemony of the travelling models industry. To do this, we propose some concepts developed from our research in the field of maternal health in West Africa, such as the ‘mechanism’ and ‘devices’ of travelling models, the distinction between ‘structural contexts’ and ‘pragmatic contexts’, and finally ‘practical norms’ and ‘professional cultures’. We do this by using various examples of the implementation of standardised interventions studied during our surveys (the partogram, focused antenatal care (FANC), prevention of mother-to-child transmission of HIV (PMTCT), performance-based financing (PBF)), which are particularly significant of how contexts strain these interventions. We rely here on research carried out for 15 years in Niger and West Africa by the Social Dynamics and Local Development Research and Study Laboratory (LASDEL; Laboratoire d’études et Recherches sur les Dynamiques Sociales et le Développement Local) on health, and more specifically, maternal health [24–30]. Since 2012, these studies have been included in an International Development Research Centre (IDRC)-funded programme on Neglected Issues Relating to African Health Systems: An Incentive for Reform, which includes a section on midwives. This research helps us understand how the multiple, unremitting standardised reforms, appearing in the form of new policies (vertical or horizontal, large scale or small scale) or new interventions or new ‘ready-to-wear’ protocols, are based on the promotion by international experts of miracle ‘mechanisms’ extracted from their emergence contexts and supposedly possessing an intrinsic effectiveness thanks to their operational ‘devices’. Also examined is how these travelling models (which always have a core component of social engineering regardless of their biomedical basis) are, when implemented in very different contexts, always confronted to selective adoption by local actors and often disguised rejections, and are generally circumvented or disarticulated.

In the absence of in-depth qualitative studies, the various ‘revenges of the context’ remain very poorly documented, and the race for new travelling models continues. Yet, as we shall see in this article’s conclusion, there are alternatives – either allowing travelling models to transform themselves according to the reactions they generate, or promoting ‘tailor-made’ reforms based on local contexts and the routine practices of health workers.

### The partogram: one case among many

The partogram is a simple, standardised tool that is presented as fundamental in the fight against maternal mortality in Africa. All schools of health in African countries introduce their students to its use, and numerous training courses are given to practising midwives.

However, in most cases in Niger (but also in neighbouring countries), the partogram is not filled out during labour, but afterwards, often at the end of the service, using standard figures. When a midwife decides to make a referral, it is generally based on her judgement alone and not on a partogram. She then fills it out before the evacuation (it must be attached to the file), taking care to enter the ‘right data’ (false, but standardised) to justify the referral.

Of course, there are midwives who fill in partograms during labour, and use them as a guide. However, according to multiple interviews with midwives conducted by LASDEL researchers, they are a very small minority. Other researchers, also working with qualitative methods and in West Africa, noted that partograms were not being filled out during labour [31, 32]. The ‘practical norm’ (see below) followed by most midwives is to fill out the partogram only after the delivery.

Although all frontline health workers (and many officials or experts) are aware of this situation, nothing is said in public settings (conferences and seminars, official publications and reports from international organisations or NGOs) about the actual use of partograms in first-level health centres and maternity hospitals. In other words, everyone speaks and acts as though partograms were used reliably and routinely, as though there were no problems surrounding their use, and as though the ‘model’ of the partogram was an effective one.

Why do Nigerian, Beninese or Malian midwives not fill in partograms during labour? Various explanations were collected from the midwives themselves during our surveys. An overload of work is the main reason given, where “*with several simultaneous deliveries, there is not enough time to fill out the partogram*” is the justification commonly given by the interested parties. However, this situation only occurs frequently in the occasional urban maternity ward, which makes the argument contestable in the vast majority of cases. The primacy granted to experience (and the resulting flair) is another, more credible, explanatory factor. We can also cite a weak culture of writing among midwives, a profound reluctance in the face of ‘bureaucratic’ tasks, a professional ethic that is often lacking, subcontracting of births to matrons, trainees or attendants, and the absence or non-functionality of the instruments (tensiometer, thermometer) needed to measure variables [33–36]. Finally, the fact that partograms are an evaluation tool during supervisions has a perverse effect [27, 31] – this encourages writing of the ‘right data’, or standard data, rather than the actual data (which could indicate failures on the part of the midwife).

In one way or another, these reasons reflect local professional contexts, which ‘bypass’ the proposed model.

The partogram is in fact an instrument of social engineering (not biomedical engineering, such as a molecule or vaccine), as is the case with many of the elements that constitute a national or international health policy. In other words, its use and effectiveness essentially depend on how it is implemented (or not) by a health system, and by frontline workers. Everything depends, finally, on the behaviour of midwives, their motivation, their competence, their professional culture and their ‘practical norms’. Furthermore, there are several other actors involved in the dissemination and the implementation (or not) of the partogram such as maternity directors (and the type of leadership they exercise), health district management teams (and their mode of supervision), partogram trainers, the health hierarchy of the national health system, and international organisations and NGOs working in the field of maternal health. Even if midwives are the first involved, the non-use of the partogram during the delivery is the result of the behaviours of all these categories of actors.

For the international (and national) experts who have developed and disseminated it throughout the world, the partogram is nevertheless a very simple tool, accessible to any health worker and usable in contexts that are poorly equipped with materials. This is what makes it valuable for the fragile health systems found in LMICs, in contrast with the sophisticated computerised monitoring of delivery rooms in the countries of the North. Its simplicity allows it to perform several functions such as following up on the delivery, aiding in decisions (referral), sharing information between health professionals, and acting as a tool for evaluating personnel and even a medico-legal document in case of deaths. In a way, it has everything a ‘miracle mechanism’ needs, at least from an armchair perspective.

The partogram’s standardisation (health workers receive ad-hoc training and receive pre-printed forms from their Ministry, UNICEF or international NGOs) and internationalisation (these trainings and forms are roughly identical throughout the world) make it a typical travelling model, among many others. Maternal health has not lacked travelling models; indeed, in the last 10 years, Niger has seen the arrival of PMTCT, emergency obstetric and neonatal care, essential obstetric and neonatal care, active management of the third phase of labour, fee exemptions for caesareans, FANC, integrated management of childhood illness, and PBF. Seen from the ground (at least in Niger), some of these models, such as PMTCT or FANC, are rather failures, some of which have been documented in yet unpublished works from LASDEL. Others have mixed results. The active management of the third phase of labour model, for example, is generally considered a success, largely because it is a medical act (injecting oxytocin) where the role of social engineering is relatively weak. However, even this model suffers from several shortcomings due to local contexts, such as delays in injection, stock shortages, cold-chain interruptions and uterine massage not being frequently performed (see Rational Pharmaceutical Management Plus Program 2007, concerning Bénin). Bottlenecks and unintended effects of travelling models in the health domain often remain to be documented in any depth, though it has been done for the exemption of user fees in Mali [37] and Niger [38], and for PBF in Bénin [39].

Health is a field where standardisation and the internationalisation of interventions, policies or procedures are very well developed. This international standardisation certainly has economic or managerial justifications, but it is also based on a belief in the intrinsic effectiveness of travelling models in the fight against disease (one of the origins of this can be found in the fight against major endemic diseases) and on an increasing ‘protocolisation’ of care, which is inseparable from the bureaucratisation of health and of all public services [40, 41]. This is largely due to the weight of the major international organisations that finance and disseminate these travelling models (WHO, UNICEF, the Global Fund to Fight AIDS, Tuberculosis and Malaria (GFATM), the Bill and Melinda Gates Foundation, etc.), although national health authorities or medical NGOs also contribute to the trend.

As with the partogram, most standardised travelling models encounter various forms of resistance from the contexts in which they are implemented. They do not ‘work’ as the experts have anticipated. This certainly does not mean that they cannot have positive effects. It would be just as absurd to say that they never work as to say that they always work. However, the problem is that quantitative tools commonly used in public health for impact evaluations and the production of evidence, such as randomised control trials (RCTs), do not reveal the implementation process [19], the unexpected effects and the strategies of stakeholders regarding standardised travelling models [42]. Although experimentalist methods have been questioned, including in the field of safe motherhood initiatives [43], they are still favoured by most decision-makers as the ideal tool for cost-effectiveness-based interventions. However, only rigorous qualitative methods can describe how standardised models are received in LMICs. When they are adopted, it is only partially, sometimes it is a step backward, sometimes it is under duress or pressure, sometimes only superficially, and rarely with enthusiasm or conviction. They are often bypassed, diverted, dismembered or disarticulated.

### The creation of a travelling model

Since the term ‘model’ has many meanings, its use here must be clearly defined. These are not computer models based on algorithms, simulations or projections, widely used in public health and in health economics. We are interested in an entirely different category of models, namely standardised intervention programmes (in this case in the health domain) aimed at inducing behavioural changes among health workers and/or patients.

We borrow the expression ‘travelling model’ from Rottenburg [44, 45]. For our part, we define it as any standardised institutional intervention, whatever the scale or field (a public policy, a programme, a reform, a project, a protocol), with a view to producing any social change, through changes in the behaviour of one or more categories of actors, and based on a ‘mechanism’ and ‘devices’ (see below) supposed to have intrinsic properties allowing this change to be induced in various implementation contexts. In the field of maternal health, these interventions aim to modify the behaviour of health workers and/or of populations.

Travelling models for reducing maternal and neonatal mortality are thus standardised interventions involving social engineering, which can have very different formats – from simple protocols or standards of care (the partogram, FANC) up to sectoral health policies (PMTCT, fee exemptions for deliveries or caesareans).

In political sciences, this travelling of models is referred to as the ‘transfer’ of a public policy. In the field of development, which is a branch of public policies developed and financed from outside sources, there are many models. In the specific field of health in LMICs, which is a sub-field of development, there are even more. Regularly replaced, they stack on top of one another; they travel a lot and they travel far.

A model always has at its root a founding experience (a success story) somewhere in the world, which international experts seize on to spread it beyond its original context. The model is manufactured around a causal mechanism that is considered by experts as an explanation for the success of the founding experience. This mechanism is supposed to guarantee the intrinsic effectiveness of the model, regardless of the new contexts in which it will be implemented. It will be disseminated in other contexts by networks combining experts and decision-makers and supported by international institutions.

In other words, the specific production of a travelling model goes through three largely overlapping main processes, namely narrative creation (a founding success story), shaping (the construction of a mechanism and its devices) and networking (global dissemination). These processes, of course, take place in an environment that public policy analyses have already abundantly described, either using a sequential perspective – emergence, formulation, decision, implementation [46, 47] – or the metaphor of ‘coupling’ between three ‘currents’ [48], namely problems, solutions and political orientations.

### The case of PBF

Take the example of PBF, also called ‘result-based financing’ (RBF) or ‘payment for performance’, which is promoted at great expense by the World Bank in all LMICs, particularly in the health sector [49, 50]. It is directly concerned with maternal health, with pregnant women and children under 5 years old being the main targets.

Like all travelling models, PBF relied on an initial success story, in this case in Great Britain. Unfortunately, the creation of a narrative for this success story through numerous laudatory publications did not take into consideration reservations or critical analyses. Indeed, several systematic reviews did not show a significant positive impact of PBF on the effectiveness and the quality of the health system in Great Britain [51, 52]. PBF was subsequently imported into Africa through a success story relay, to Rwanda in this case [53–57]. The Rwandan PBF, experimented with in 2002 and generalised in 2006, has been widely presented as a model for Africa, including in such a prestigious journal as *The Lancet* [58]. However, critical analyses were also ignored in the Rwanda case, such as in the study by Kalk et al. [55].

A travelling model must refer to an exemplary certified experience somewhere in the world. This is an essential step for its production and exportation. However, a local experience, with its context, its originality, its limits and its specificities, does not become an exportable model by itself, neither by the sole virtue of its success or its reported good results. Experts must extract from this experience the explanatory ‘mechanism’ for its effectiveness, and translate this mechanism into operational devices. The PBF mechanism is simple in principle, it relies on the indexing of the salary of health professionals on their performance (number of acts and quality of acts). In other words, it determines indicators allowing those who work more and better to earn more. The partogram mechanism is also simple, it advocates the manual measurement of variables and their recording on a pre-printed diagram that allows alert thresholds to be defined. However, the operationalisation of a mechanism is always complex. The mechanism must be translated into intervention ‘devices’, which are part of social engineering and go through multiple ‘instruments’ [59, 60]. ‘Devices’ refers to the fundamental organisational components and the operational, technical and institutional measures that enable a mechanism to be implemented within a public health policy or intervention. In the case of PBF, the devices are sophisticated. For example, in Burkina Faso there are 102 performance indicators for health workers that PBF programme coordination must compile and monitor, not to mention the 61 indicators of the World Bank-funded basic evaluation. For first-level health centres alone, no fewer than 23 quantitative indicators and 11 qualitative indicators were developed by the experts.

A mechanism does not appear fully formed in the minds of experts; they elaborate it using an operation that simplifies and ‘reduces’ inaugural experiences. This is what Ancelovici and Jenson [61] call the ‘decontextualisation’ phase. In the lessons learned from the reference success stories, a particularly complex reality must be divided into two parts – a ‘mechanism’ with intrinsic effectiveness and a ‘context’ that is relegated to the status of adjuvant. This distinction is not spontaneously given but, rather, constructed. The complexity and diversity of the initial success stories were ‘reduced’ to the supposed effectiveness of an explanatory ‘mechanism’, which would therefore be exportable. The PBF mechanism could be ‘produced’ only by extracting it from what was simultaneously ‘produced’ as its emergence context, whether in Great Britain or Rwanda. For example, in the case of Rwanda, many contextual elements were decisive in the ‘success’ of the implementation of PBF, namely the very specific political situation, the very strong social control that prevails, the regime’s capacity to ensure relatively satisfactory functioning of public services, less widespread and less visible corruption than elsewhere, and the effective use of sanctions within the administration and the fear they arouse. These elements contrast strongly with the situation faced by most health systems in African countries, including ‘every-man-for-himself-ism’ and weakness of the State, poor functioning of public services, open and widespread corruption, and impunity, among other issues [62]. The reduction of a success story to an effective mechanism involves excluding a whole series of other potentially explanatory co-factors of this reference success story, which are relegated to the status of contextual elements that no longer need to be considered.

The reduction to a mechanism goes together with its construction as the core of the new model (comparable to the core of a reactor). An argument of justification and legitimation is established, serving also as a sales pitch for the mechanism. The success stories at the origin of the travelling model must be validated; the mechanism must be certified and celebrated. PBF thus draws on considerable literature and on an impressive mass of evaluations, seminars, workshops, training courses, study tours, reports and manuals, which requires a sizable specialised workforce. Advocacy networks, expert networks and funding networks must be set up. For PBF, the World Bank, WHO, the GFATM and various other agencies and countries (particularly Norway) have collaborated, for example, in structures such as Inter-Agency Working Groups on Results-Based Financing or Health Results Innovation Trust Fund [55]. In this process of promoting a travelling model, there is no consideration for negative or even just reserved evaluations highlighting unexpected effects. For instance an overview of research on the effects of RBF in LMICs [63] was ignored, although it reaches three significant conclusions confirmed by various subsequent studies. Firstly, “*There are few rigorous studies of RBF and overall the evidence of its effects is weak*” (especially in the long run); second, “*The use of RBF in LMICs has commonly been as part of a package that may include increased funding, technical support, training, changes in management, and new information systems. It is not possible to disentangle the effects of RBF and there is very limited quantitative evidence of RBF per se having an effect*”; and thirdly, “*RBF can have undesirable effects, including motivating unintended behaviours, resulting in distortions (ignoring important tasks that are not rewarded with incentives), gaming (improving or cheating on reporting rather than improving performance), corruption, cherry-picking patients that make it easier to reach targets and earn bonuses and selecting out more difficult patients, widening the resource gap between rich and poor, dependency on financial incentives, demoralisation due to feelings of injustice, and bureaucratization.*” ([63], p. 4–5.) While the first two conclusions point to the difficulty of isolating and evaluating the intrinsic effectiveness of a mechanism, the third highlights the unexpected effects of implementation contexts.

These phenomena are not specific to health; they are found in all areas of development, as illustrated by the cases of micro-credit, based on the popularisation of the Grameen Bank's experience in Bangladesh [64], or cash transfers, based on the popularisation of the experiences of Bolsa Familia in Brazil and Oportunidades in Mexico [65]. This last case provided the basic arguments for the analytical framework developed here.

### On the concept of ‘mechanism’

The production of a mechanism corresponds to a certain ‘theorisation’, highlighting a supposedly reproducible causal chain. Our definition of the mechanism corresponds, in part, to what some specialists in programme evaluation – ‘theory based evaluation’ [66–68] – call ‘program theory’ or ‘logic of intervention’. In other words, a “*set of hypotheses that explains how and why the intervention is expected to produce its effects*” [69]. However, in our view, the mechanism is not constructed solely of hypotheses (or ideas, words or discourse); it is also, or above all, an institutional and organisational arrangement around these hypotheses, in other words, a set of ‘devices’ commonly called the core components of a programme.

It is the ‘mechanism plus devices’ unit that defines the heart of a travelling model.

Our definition of ‘mechanism’ is thus distinguished from a recent meaning of ‘mechanism’ in the social sciences, following Elster [70] and Hedström and Swedberg [71, 72], where it refers to a causal process that is ‘real’, but concealed, and must be deduced/unveiled/constructed by the social sciences [73]. For our part, a mechanism is not a real but hidden causal process, but rather an alleged causal process explicitly postulated by a standardised intervention model.

At first view, our definition seems to be closer to ‘realistic’ evaluation approaches [74], which rightly insist that the outcomes (O) of an intervention (of a public policy, a programme) depend on the interactions between the intervention context (C) and the intervention mechanism (M); they are therefore interested in the ‘CMO’ configuration. We share their concern with considering the context as a decisive element in any implementation of a programme or public policy (and thus of a travelling model). However, there is a significant lack of clarity among authors who adhere to this approach regarding the exact content of the ‘mechanism’ and the conceptual or empirical differentiation between the mechanism and the context [16, 42, 69, 75–77]. According to the most common definition, following Pawson and Tilley [74], the mechanism of an intervention would consist of the “*ideas and reactions of the actors involved in the intervention*” [69], a proposal that remains enigmatic, to say the least. For us, this confusion around what a mechanism would be is linked to the choice of making the intervention mechanism a ‘real’, but hidden, causality, based on the reactions of the actors to the intervention, and which would, beside the context and through it, be at the origin of the effects of the intervention (outcomes). After reviewing the variety of uses of the concept of mechanism in realistic approaches to evaluation, Lacouture et al. [69] propose a definition that does nothing to dissipate the prevailing confusion: “*A mechanism is an element of reasoning and reactions of (an) individual or collective agent(s) in regard of the resources available in a given context to bring about changes through the implementation of an intervention*.” This acceptance makes it necessary to distinguish between the actual intervention and the explanatory mechanism, which produces ‘real’ effects, a mechanism which the researcher would be required to reveal (but which in fact remains a ‘black box’). It also forces the actors to be placed on the side of the mechanism rather than on the side of the context, which is difficult to sustain. It seems much simpler not to venture into this and to consider the mechanism as the ‘operational theory’ of the intervention, put into practice through the devices. Equally important are the perceptions and reactions of the actors involved in, or targeted by, the implementation of a travelling model, which must be considered as central elements of the implementation contexts.

The distinction between a mechanism and a context is therefore not in our view a scientific operation carried out by a researcher a posteriori, it is a deliberate social construction at the heart of the production of travelling models, according to which experts distinguish between (1) what would be an explanatory variable (the mechanism), which is their business and around which they construct their interventions (through devices), and (2) what would be contextual variables (including local actors’ routines), the management of which remains problematic and uncertain. The construction of a mechanism is inseparable from the language of variables (and, within this language, the promotion of an ‘explanatory variable’).

### Travels of the model and the revenge of the contexts

The mechanism and its devices are then deployed in new contexts from this point. For this, they must pass through informal and formal networks that will allow the model to travel by justifying it, legitimising it scientifically and promoting it. The sociology of science and technology promoted by Callon and Latour [78], under the name of sociology of translation or actor-network theory, has focused specifically on the role of these networks such as forming alliances, ‘enlisting’ supporters, etc. The replication of a model ‘far from the origin’, and thus far from the initial success story, is not spontaneous. It is based on an alliance (a coalition) of experts and decision-makers who make themselves the ‘social bearers’ of what might be called the ‘idea’ of the travelling model (its mechanism), its narrative and its promotion, to the point where its importation is put on the agenda in various distant countries, generally through the conjunction of international financing and/or technical assistance.

International institutions play a central role as ‘travel agencies’ for models, particularly in the field of health; however, they are not the sole actors – social engineering consultants, think tanks, communities of practice and medical NGOs are also involved. These institutions justify their interventions based on the intrinsic effectiveness of the model they promote, and tend to ignore or minimise the unexpected effects of its implementation in complex contexts little known in the world of the experts and decision-makers.

### The test of contexts: the case of prenatal consultations

The concept of the ‘test’ (or *épreuve*) is an important component in recent Francophone works in social sciences, between pragmatic sociology and the sociology of science and technology [79–81]. The test of implementing a travelling model in contexts far from the original success story is a major challenge, which we are surprised to find so underestimated at this point. Contrary to the accepted notion among experts constructing and disseminating models, the intrinsic effectiveness of a model is less important than the implementation contexts, and more specifically, the routines and constraints of the health personnel in contact with the users. These frontline workers will be the ones who test the proposed model (which is in general, imposed by the health hierarchy), adapt it to their working conditions and professional culture, and accommodate it in their own way, often by bypassing it, diverting it or dismantling it, and sometimes by ignoring it or boycotting it de facto.

Let us take another example. Antenatal care (ANC) carried out in first-level health centres by midwives in West African countries, inspired by an old European model from the early 20th century, is considered a key element in the fight against maternal mortality. Nevertheless, the classical model as it has long been implemented in Niger and in neighbouring countries suffers from various shortcomings. As revealed by our inquiries, most of the time, fast and sloppy ANC does not fulfil its primary function, which is to detect pregnancies at risk in order to prevent them or to plan a delivery in a health facility equipped for obstructed deliveries. For instance, a previous study on 330 pregnant women has concluded that “*in Niger, the quality of the screening* [by midwives] *for risk factors was poor*” [82]. A new travelling model of ANC was therefore adopted by WHO in the early 2000s [83] and has been widely disseminated in Africa, that of focused ante natal care (FANC). FANC is based on a pre-printed form that includes a series of items to be scrupulously investigated and checked off, thus enabling a midwife, following the print-out to the letter, to perform the battery of operations required for an effective and personalised FANC (observations, examinations, questions, explanations). Based on the answers to a first set of questions and observations, the files are divided into two categories, one for pregnancies without specific risks (basic components) that will be followed per a standard protocol, the other for at-risk pregnancies (specialised components) that will receive a specific follow-up. The principle of this mechanism is the same as that of the partogram (self-monitoring guided by tools to be filled out in writing), but it rests on more complex devices (many more items, biological examinations, sustained dialogue with the parturient, two types of files, etc.). The promotional argument is equal to that for the partogram – it is a simple tool adapted to the deprived conditions of LMICs: “*FANC is the best approach for resource-limited countries where health professionals are few and health infrastructures are limited*” [84]. However, once again, the implementation contexts fail to line up with the expert-generated scenario.

The problem of time management arose very quickly upon the arrival of FANC in Niger and other African countries [85, 86]. In fact, a minimum of 40 minutes is required to complete all the items on the form during the first visit (according to WHO). However, ANC, which previously took less than 15 minutes on average, was already largely attended by pregnant women, and so waits became much longer. This was made worse by the fact that, in most Nigerian health facilities, only two mornings per week are devoted to this, often with only one midwife staffing the activity. This obviously poses a problem for the routine organisation of activities in primary level health centres. Added to this is the strong reluctance and uneasiness of midwives concerning the bureaucratic tasks and the writing. Under such conditions, going from about 10 minutes for ANC to 45 minutes for FANC is an impossible mission. Most midwives, therefore, do not perform a real FANC, except when a supervisory team passes by. They do not document many items, and/or fill out the forms in a standardised way. Some constants are rarely taken (such as blood pressure and ventricular tachycardia) and the speculum is not used (it is usually absent in many maternity units). The delivery plan and potential complications are not discussed.

Ignorance of the problem posed by the length of the FANC session, ignorance of how work is organised in health centres, and ignorance of the professional culture of midwives, are direct causes of the failure (or partial failure) of FANC. Information on the actual non-delivery of FANC rarely gets back through the monitoring and reporting systems of the Ministry of Health and the vertical programmes set up by international institutions, and therefore no rectifications are made. No one in the health hierarchy presents the problem in public, even if it is known in private. The laudatory statistics that circulate on FANC mainly involve training (number of training sessions, number of health workers trained, number of post-training follow-ups), FANC activities reported (number of FANC performed, coverage rates) or logistical and financial aspects (number of forms disseminated, rate of budget execution). They do not concern the quality of the FANC that is supposed to have been performed.

### On the concept of ‘context’: pragmatic contexts and actors’ roles

Maternal health models introduced in LMICs rely first and foremost on the intrinsic attributes of their mechanism and therefore neglect the massive existence within health systems of an entire series of elements related to the behaviours of the actors and to local contexts. However, these elements have been emphasised in some works, including strategies for securing aid [87], corruption [88, 89], clientelism [90], local professional routines, absenteeism at work, informal earnings [91, 92], types of leadership, management and organisation of work [93], interventionism regarding posting and transfer [94, 95], and the opportunistic fabrication of figures or biased statistics [96, 97], to name a few. More generally Greenhalgh et al. [98] highlight, “*the important principle that the attributes* [of an innovation implemented in health services] *are neither stable features of the innovation nor sure determinants of their adoption or assimilation. Rather it is the interaction among the innovation, the intended adopters and a particular context that determines the adoption rate*”.

It is true that, within public health, contexts are not unknown. Nevertheless, they are reduced to a set of so-called contextual sociodemographic, institutional or epidemiological variables (rates, indices and other indicators relating to income, health coverage, access to care, the poverty line, education, health system, use of public services, GDP, HDI, etc.), which must be quantified and can be aggregated, weighted and enumerated in countless variations. For example, in a review of the literature on the role of contexts in programmes to improve the quality of care [99], the authors, after distinguishing some families of contextual variables such as ‘outer setting’ (economic, social and political environment), ‘organisational setting’, ‘individuals and their roles’ and ‘networks’, identified 66 variables that were measured. This logic of variables, its inflationary drift and the quantitative obsession (no qualitative study was considered in this review) therefore completely remove its meaning from the concept of context as we understand it. It leads to ignoring or minimising what constitutes the decisive element of the contexts, namely the actors. As Abbott [100] has pointed out, “*in the causal modeling tradition, variables and not actors do the acting*” (cited in Hedström and Swedberg [72]). On the contrary, the actor must be placed in the centre of the contexts (and not treated as one of many variables).

Admittedly, the concept of context itself can seem ambiguous and polysemous. Firstly, from the perspective of science studies, every action at any level takes place within a context, each of which is specific. The construction of a mechanism or the global dissemination of a model also takes place within contexts, and the offices of the World Bank or those of any Ministry of Health are as much contexts as a rural maternity hospital or a hospital consultation. They all merit the researchers’ attention. Admittedly, these are very different contexts. The contexts of think tanks or meetings of high level experts (contexts of narrative creation, shaping or networking of a public policy) are not the same as those in the poor districts of Dakar or the villages of Niger (implementation contexts). For the purposes of this paper, we reserve the term ‘context’ only for the implementation contexts (national, regional, local) of a travelling model.

We therefore propose a more differentiated view of the implementation contexts, distinguishing between structural contexts (in the background) and pragmatic contexts (in the foreground). At the centre of the pragmatic contexts is the role of the actors, which means accounting for the concept of agency developed by Giddens [101], widely used in the social sciences today and imported into the field of development by Long [102]. Defined in other words, it is the cognitive and strategic ‘room for manoeuvre’ of the actors (their ability to know and act relatively autonomously), and the norms and constraints within which they operate [103]. Structural contexts (generally characterised as being linked to the economic, political, social or cultural environment) only affect the importation of a travelling model through pragmatic contexts, in other words the latitude of actors (or stakeholders) and their interactions. Structural contexts are available constraints and resources that act to influence the representations and practices of the strategic groups involved in the implementation of public health travelling models (national experts and policymakers, public and private health professionals, NGOs, local authorities, beneficiaries, non-beneficiaries), which constitute the pragmatic contexts and are at the heart of what we call the revenge of the contexts.

Structural contexts are typically described through the language of variables, figures and standardised quantitative indicators. Conversely, pragmatic contexts are more acutely described through qualitative approaches such as narratives, process analysis, case studies or interviews of stakeholders, and the use of concepts such as actors’ logics, actors’ perceptions, practical norms, professional cultures, local cultures, strategic groups and more.

In other words, social actors are at the core of implementation contexts (seen as pragmatic contexts), with their networks, their interactions, their informal rules, their organisational routines, their strategies and their motivations. This perspective is clearly distinct from the language of variables. Putting actors’ behaviours at the centre of context analysis requires new conceptual tools.

### Gaps and practical norms

This central role of actors, their perceptions and their strategies, which are always far from the expectations or the presuppositions of experts, introduces two types of gaps, namely an implementation gap and a normative gap.

#### Implementation gap

The implementation gap is an inevitable process. As well-prepared as public policies may be, when implemented, they inevitably experience discrepancies between what is expected and what happens on the ground. In other words, “*differences between their formal objectives and goals and those that emerge through the practices and strategies pursued by the actors at different organisational levels … The relation of policy and practice is not an instrumental or scripted translation of ideas into reality but a messy free-for-all in which processes are often uncontrollable and results uncertain*” [104]. This eloquent title “Good on paper: the gap between programme theory and real-world context,” which introduces a revealing analysis of the implementation gap of a community midwife programme in Pakistan [22], could be applied to all travelling public health models.

#### Normative gap

A normative gap is the difference between the official rules and procedures incorporated in a travelling model and the various norms, habits and routines in effect in the implementation context. Any standardised public health intervention includes a set of new formal norms developed by international, and sometimes national, experts to make the intervention mechanism efficient. However, these imported norms are distinct from the social or informal norms that regulate the behaviour of the different actors affected by the intervention.

In fact, at least two main types of normative registers can be distinguished within the implementation contexts of health interventions, namely the social norms of users (patients, pregnant women, communities) and the practical norms of health personnel (nurses, midwives, doctors).

##### Social norms of patients

The social norms of the beneficiary populations are very complex and diverse and can in no way be reduced to a few stereotypes. Public health sometimes tends to reduce non-Western local cultures to a few more-or-less-exotic ‘traditional’ clichés, in other words, a ‘culturalist’ drift [105, 106]. In particular, social norms relate to illness, suffering, the search for care, modesty, decorum, mutual aid, collective action, gender relations, patron/client relations, education, the supernatural, death, relationships with the State, and so on. These social norms, or local cultures, vary widely from one site to another. They are, in fact, underestimated by experts in travelling models (who nevertheless recognise their existence on a rhetorical level).

##### Practical norms of health workers

On the other hand, there is another type of contextual norm, even more unrecognised by standardised interventions, one that regulates the discrepancies between the official norms and the routine practices of health workers. Health workers are, in fact, far from always following the professional and ethical rules that they have been taught. These ‘non-compliant’ behaviours are not random; they are relatively predictable and routinised, and in fact follow latent and implicit norms that we call practical norms. This highly pragmatic exploratory concept of practical norms is distinguished from other interesting attempts to analyse non-compliant behaviours of health workers in the face of bureaucratic interventions and clinical guidelines, including in northern countries. For example, relying on United Kingdom data, Checkland, Harrison and Marshall [107] emphasise the importance of contexts and the impasse represented by the dominant approach of so called evidence-based medicine, based on the identification of barriers of change, dissemination of clinical guidelines and RCTs (see also Greenhalgh et al. [98]). However, they prematurely favour the explanatory concepts of collective identity and sense-making imported from social psychology. These, according to our experience in Niger, are far from being the dominant factors in the non-compliant practices of health workers.

The concept of practical norms appears a very useful tool for empirically analysing how the non-compliant behaviours of health workers are regulated, particularly in their deviations from the injunctions of travelling models. Simply invoking the importance of pragmatic contexts regarding standardised interventions is not enough. It is necessary to have tools to analyse these contexts.

We start from this first observation, repeated many times in our surveys, namely that the behaviours of health personnel in Niger, as in French-speaking Africa, fall far short of the official norms established by the Ministry of Health or continually introduced by travelling models. Yet, they have been trained to observe these norms, and regularly recycled through expensive training, so it is not a matter of ignorance.

A second observation must be considered, namely that their ‘non-compliant’ behaviours are not random or anarchic. They are largely predictable for those familiar with the local professional context (colleagues or users). They form the framework of the local professional culture [108]. In other words, these behaviours are shared and convergent (although there are, of course, exceptions). They are regulated in a latent, underground, tacit manner in their deviations from the prescribed norms.

We call these implicit regulations ‘practical norms’. This concept has already been used to describe the routine behaviours of midwives in Senegal [109] or Niger [108], to study the functioning of emergency rooms in Niamey [110] or a hospital department in Soweto [111]. For a detailed theoretical presentation of the concept of practical norms and its possible uses, see Olivier de Sardan [112] and De Herdt et al. [113]. To examine the practical norms of health workers requires that we redefine their professional cultures – a professional culture is not simply a collection of knowledge and ‘good practices’ learned during a training course; it is also a set of ‘tricks’ learned on the job to accomplish their daily work by departing from this knowledge and these good practices. It is a combination of official and practical norms.

#### The professional culture of midwives

Let us take the profession of midwife in Niger – it is characterised by a series of practical norms. Here is a list, in no particular order, of all of the practical norms confirmed by the health professionals gathered at the LASDEL Vocational School for maternal health personnel held in Niamey in October 2015. The formulations are our responsibility (they are never publicly expressed as such and most often remain implicit), but we have encountered each of them repeatedly in our survey sites in Niger over 15 years, through multiple observations, case studies and private interviews.

- A midwife is more competent regarding deliveries than a public health doctor and knows better what to do.
- The management of a delivery is a matter of experience and flair.
- The maternity hospital is a space that ‘belongs’ to midwives, not to parturients.
- Eutocic deliveries (without problems) may be delegated to matrons, trainees, midwife assistants or attendants.
- Bush women and young parturients are ignorant and impatient.
- Women with ‘false labour’ (insufficient dilation) disturb the department and must be sent home if possible.
- When a woman in labour does not ‘push’ enough, she must be compelled to do so by any means (insults, harsh treatment, or threats of referral are legitimate and for her own good).
- A ‘recommended’ parturient deserves attention and consideration, which does not need to be given to anonymous parturients.
- Bureaucratic tasks are unnecessarily time-consuming chores.
- Sanctioning a midwife is not proper; it would be wicked.
- It is normal to arrive to work around 9 a.m. and to leave around 1 p.m.
- It is legitimate to skip work for a social ceremony (baptism, marriage or death of a relative or of a simple acquaintance).
- Seeking ‘informal’ earnings at the expense of parturients (sales of products, charging for free acts, etc.) is normal.
- It is necessary to go to continuous training regardless of the content (to receive per diems and improve the CV).
- The intervention of highly-placed acquaintances is needed to obtain interesting assignments.
- Any additional task deserves a monetary bonus.
- Any funding from donors is an opportunity to ‘take one’s share’ in passing.
- Everyone must manage their activities without interfering with those of others.
- The bureaucratic tools must be filled out with standard data, regardless of the actual data.
- Staff meetings are a waste of time.
- In these meetings, one should not point out individual failures.
- If small equipment is missing, wait for the hierarchy to replace it.
- Faced with the recurring breakage of certain products, it is necessary to ‘make do,’ making a substitute if possible (for example, midwives use saline in place of the solvent to perform the PMTCT test, or make an eye wash for newborns with a mixture of Betadine and saline (50% of each solution).

These practical norms are of course never publicly stated as such, contrary to official norms (explicit through regulations, procedures and training) and social norms (explicit through family or religious education and social interactions). However, they are at the heart of Nigerian maternity routines. They constitute the more-or-less hidden basis of the professional culture of midwives and obstetrical workers. They regulate the response of midwives to a travelling model and most often explain its failure, partial or total.

Only a few writings in medical anthropology (because they have access to qualitative methods, prolonged observation, insertion in the environment) have empirically documented the ‘real’ or ‘non-compliant’, everyday behaviours of midwives in Niger or other West-African countries [27, 28, 31–35, 108, 109, 114–119]. Otherwise, there are no numbers and no statistics. Evidence in supervisory reports is little to none. Annual activity plans for districts and national health development plans never mention them in the sections on combatting maternal mortality. They are also rarely mentioned in public health literature (and when mentioned, they are summary, partial and euphemistic references). In other words, the maternities spoken of in the world of public health are largely fictitious, paper maternities, far removed from the realities experienced by the parturients. Travelling models address these paper maternities, and are built on their image.

### Why this amazing recurrence of travelling models? Governance of global health

Of course, even within the major international organisations, although they are major generators of travelling models, there are various statements warning against standardised regulations and underestimating contexts, as stated in a WHO publication: “*Considerable caution is needed in scrutinising formal management structures, rules and regulations, as they may bear no resemblance with actual management practice*” [120]. More broadly, the standardisation of development policies has already known longstanding criticism from the social sciences, whether emanating from the political economy, from Hirschman [121], a pioneer, up to Naudet [122] and Easterly [123], or in the field of development anthropology from numerous authors [124–127].

Despite this, travelling models continue to prosper and grow under various names rather than decrease in number or volume. The form of governance specific to global health (the weight of international organisations, private foundations, laboratories and NGOs) favours so-called high-impact interventions. The social sciences therefore have very little influence in this field. There is even sometimes the feeling that any critique of a travelling model only creates the conditions for another model to take its place, “*Better theory, new paradigms and alternative frameworks are constantly needed …, strangely little attention is given to the relationship between these models and the practices and events that they are expected to generate or legitimise in particular contexts*” [124].

What might the reasons be for such perseverance in the incessant production of travelling models? We will mention five here.

#### A job market for experts

“*Despite the failure of these systematic transfers of models from the North to Africa since independence, they continue because experts and consultants from the North and South, professionals from the South, and leaders from the North and the South benefit from them*” [128]. The production and circulation of travelling models is in some ways the core business of international public health institutions and employs hordes of staff, from the design (public employees, researchers and international experts) to implementation in the field (international and national consultants, trainers, NGO agents, health workers). Each new model has its own market of specialists and practitioners, both nationally and internationally.

#### Institutional routines

The weight of path dependency cannot be underestimated either. Consultancy offices, public health departments, development agencies, international organisations, major foundations and large medical NGOs have, for several decades, developed technical and accounting procedures and know-how essential to the manufacture and dissemination of travelling models such as creating narratives and telling success stories, dressing up and legitimising mechanisms, devising devices, procedures, seminars, guides to good practices, etc. We can also add the design and use of standardised tools for planning (logical framework) and evaluation (RCTs) that are particularly suited to travelling models [43] in that they focus on the efficiency of the mechanisms and systematically ‘neutralise’ the role of contexts. The production and dissemination of travelling models have become routine practice for many institutions. We find here what Di Maggio and Powell [129] called the isomorphism of organisations that, in a given field, tend to behave identically. Travelling models also have a structural affinity with ‘command and control’ approaches, scaling up strategies, and (statistical) evidence-based policies, which are at the core of the organisational cultures and professional competencies of public health institutions.

#### Supply-driven health policies

Public policies in the field of development generally correspond to the ‘garbage can’ ideal types [130]. As noted by Naudet [122] on development policies and programmes, it is a matter of “*finding problems for solutions*”. This strong supply-driven policy, based on models developed by international institutions, faces a weak (and sometimes non-existent) ‘demand policy’ from the Southern authorities, who are mostly followers and conformists, preoccupied above all by the resources (income) that external actors will provide, and little concerned with putting at the forefront the contextual concerns that they should theoretically carry. The rhetoric of good practice illustrates this, it is essentially practices complying with the norms promoted by travelling models, from a ‘good student’ perspective. This is one of the perverse effects of aid dependency (or ‘development rent’) [131]. There is little or no pressure on the demand side to offset or alter the incessant and pressing supply of travelling models. In this respect, responsibility for the hegemony of travelling models cannot be attributed solely to experts and international donors. Governments and experts from countries in the South are largely responsible for this.

#### Funding rationales

It is much more difficult to obtain modest funding for innovative experiments grounded in local contexts than significant funding for travelling models; all public health professionals (and all development professionals) confirm this. The editing and format of the files, the financing mechanisms, the disbursement procedures, the management methods and the audit practices all contribute to giving priority to exportable mechanisms and devices that allow economies of scale and can be evaluated using quantitative criteria.

#### Biomedical software rooted in mentalities

Belief – because this is what it is, after all – in the intrinsic effectiveness of a mechanism placed at the centre of a public health policy rests on an experimentalist and technical basis that has proved its worth in certain areas. A new molecule carries such intrinsic effectiveness, with a universal vocation, and, from a purely therapeutic point of view, can be applied in the most diverse contexts. It is therefore tempting to believe that the same is true of the mechanisms placed at the centre of social or institutional technologies and to credit them with the same potential effectiveness as biomedical (or industrial) technologies. RCTs are based on such an extrapolation.

It is a very widespread assumption to believe that what is true of biomedical engineering would also be true of social engineering. Nothing could be more false. The transition from biomedical engineering to social engineering is a radical change of world of reference. In the social world, the classic experimentalist statement ‘all other things being equal’ has no meaning; its implementation is impossible [132, 133]. The multiplicity, complexity and interweaving of the variables involved in any social intervention, no matter how small, makes any attempt to reduce it to a single explanatory variable illusory. Nevertheless, this illusion persists in certain sectors of the social sciences governed by a positivist vision of the world. It is especially dominant in health policy development and implementation bodies.

The case of vaccination campaigns clearly shows the gap between biomedical engineering and social engineering, because they combine the two dimensions, as many interventions in public health do; the effectiveness of the one contrasts with the setbacks of the other. While a vaccine shows intrinsic properties that are essentially context independent (the same polio vaccine protects a Wall Street manager or a farmer in the Mekong valley), its administration is based on institutional architectures and organisational arrangements that are entirely social and profoundly context dependent. Take for example, National Immunisation Days, a widely celebrated travelling model, organised and financed at great expense each year by Gavi in all African countries. It is presented worldwide as a great success based on statistics showing a dramatic increase in vaccination coverage. In fact, it has many perverse effects [97], due in part to the fact that they have been functioning for years on a PBF-type mechanism (paying for the vaccinations based on performance). This results in disorganisation of health services (health workers abandon routine activities to run after premiums), systematic multi-vaccinations and falsification of figures (to inflate the results), and false missions of executives (linked to income-appropriation strategies by the health hierarchy; for Niger, this aspect was highlighted by an international audit that resulted in the temporary incarceration of dozens of doctors [134]). Various LASDEL qualitative surveys not yet published attest to the importance of multi-vaccinations and falsification of figures. Further, several more conventional public health studies also show the limitations of these vaccination strategies, including in Burkina Faso [135], and evoke ‘the fallacy of coverage’ [136].

### Starting from practical norms to bring about changes: the quest for innovations and reformers from within

What are the alternatives to travelling models? This domain remains very poorly known and has yet to be explored systematically and empirically.

One possible avenue is to begin with practical norms, introducing gradual changes in them rather than importing new standardised official norms over and over during successive interventions that stack on top of each other without changing routine behaviours and without credible strategies of appropriation and sustainability. Which of the practical norms currently in maternity hospitals can be amended, modified, improved or even enhanced? Which ones must and can be abandoned, and how, to the benefit of which others? How can better practical norms be introduced and made sustainable and institutionalised? This perspective favours ‘tailor-made’ over ‘ready-to-wear’. Furthermore, practical norms can also sometimes be positive and innovative. They are not always practiced for opportunistic, conformist or selfish purposes and can, on the contrary, make it possible to manage or tinker solutions, invent palliative practices, develop coping strategies that benefit patients, manage the shortage or lack of motivation in colleagues, try adapted forms of leadership, and so on.

We have met with few ‘reformer’ midwives or gynaecologists who are trying to improve the quality of health services based on real contexts and problems in the field, without fully applying standardised interventions, which is far from strict compliance with travelling models. Their initiatives are discrete, scattered and largely invisible. They deserve to be known and documented.

#### The case of a reformer midwife

To give an example, we will use a case study concerning a ‘reformer’ maternity hospital director in Niger [119]. This survey was carried out by Maman Sani Souley Issoufou as part of a master’s thesis in medical anthropology from Abdou Moumouni University and of the research programme on midwives financed by the IDRC and led by Aïssa Diarra. This director succeeded in modifying the practical norms of the personnel in her maternity hospital on three points, without using the official norms (and in fact, maintaining a separation from them).

##### Lateness

The official working hours of health workers is 7:30 a.m. to 4:30 p.m. with a one-hour lunch break. But the midwives routinely arrive around 9 a.m. and leave at about 1 p.m. (except when on call). To try to enforce schedules, the Ministry recently attempted to intervene; it requires the health units to keep an attendance book, with a red line at 8:00 a.m. It is not surprising that staff everywhere continue to arrive as usual around 9 a.m., but enter a fictitious arrival time before 8 a.m. in the book. The director of the Salam* (name has been changed) maternity hospital does not use the official attendance book; she knows it is useless. On the other hand, she herself arrives at 8 a.m., and then goes around all the departments to greet the staff, who feel compelled to be present. The director, because she sets an example, and by exerting a ‘soft’ control, does better than the bureaucratic measure officially imposed by the Ministry, but without respecting the official norms (beginning at 7:30 a.m., using the notebook).

##### Haemorrhages

Postpartum haemorrhage is most often the fault of the midwife in charge of the delivery, but, in Niger, no one uses the legal sanctions for professional misconduct. The director of the Salam maternity hospital does not use legal sanctions either. However, she has ‘invented’ a local sanction in the form of a practical norm that she has imposed, wherein, in case of haemorrhage, the midwife must personally accompany the parturient to the referral centre. For the midwife in question, this is a waste of time (and money) and, worse, a disgrace – the reference centre agents know that there is fault if they are brought a haemorrhage from a maternity, and see the culprit before them.

##### Informal payments

Midwives (and other maternity staff) earn an estimated average of US$10 per delivery from informal (and illegal) payments that they demand from the parturients (sale of oxytocin, serum, suture thread, gloves, etc.). The director of the Salam maternity hospital did not attempt to remove these levies (which would be unrealistic), but rather to reduce them. She assembled her staff, setting a limit of US$5.

Here we have an example of local reforms improving the quality of service delivery by building on existing practical norms, albeit by modifying some of them, yet without attempting to enforce official norms, which are not only currently not applied (revenge of the contexts), but inapplicable (without a radical reform of the civil service and the State).

## Conclusion

In Niger – as in all countries – there are certainly other reformer midwives (or, more generally, reformer health personnel). They currently constitute an invisible minority, but why not try to network them, and rely on them to define various ways of improving maternity hospitals? This is what is being attempted by a LASDEL action research programme, entitled “Maternal and adolescent health in West Africa: Toward low-cost reforms grounded in reality” covering Benin and Niger and funded by the IDRC. The central component of the programme is researching and documenting local reforms and pockets of effectiveness [137–139] within the maternal health system. In other words, formal or informal innovations that have led to behavioural changes in favour of a better quality of care. A dozen or so reformer midwives, calling on their experience in the real world setting and their knowledge of the practical norms in use, will be asked to define themselves experimental actions that they will implement in a few maternity hospitals, with the ultimate objective of acting as support to local and regional ‘reformer coalitions’ in the field of maternal health, in order to modify and improve, step by step, the professional culture of Nigerien midwives (based on practical norms). It means facing very concrete challenges, what to do with partograms, considering what happens in reality, or with FANC? Modify them, get rid of them, replace them, and if so, by what? Relevant answers can be provided only by field experts, i.e. reformer front-line workers, not by armchair experts.

To transform these professional cultures, there are certainly other avenues than the one we are suggesting here. There are other ways of starting from contexts. For example, alongside these silent ‘reformers from within’ who operate individually, some institutions, sometimes originating from without and usually small, are exploring innovative approaches based on local realities. One example is the case of the Network of Safety approach in maternal health, carried out by an NGO (Organization of Human Welfare), which is based on the following concern: “*Our argument is that cultural specificity is irreducible and not standardisable, but rather a basis for creating plural and complex models for intervention. How, then, might we make use of cultural specificity as a starting point for designing interventions, rather than as a reproducible and reducible set of variables that would look structurally the same in any target community?*” [140].

These attempts at context-based reforms or changing behaviour from practical norms have demonstrated a dramatic knowledge gap. Empirically documenting and identifying all ‘low noise’ (or even silent) innovations taking place within health systems and in the real world is one of the priority tasks that can be assigned to medical anthropology. It is regrettable that there is very little interest in public health professional journals for such an approach, due to the major hegemony of travelling models and vertical interventions and the great weight of the quantitative methods associated with them [43, 141]. There are also very few credits available for such research and experiments, although their funding needs are insignificant compared to the financing required for travelling models or randomised impact studies. Finally, there is very little room or audience for them in conferences on public and global health, or even on health systems and policies. Fortunately, there are few professionals within public health institutions (including within the Safe motherhood initiative) who wish to move in this direction and call for ‘epistemological diversity’ [43]. In this respect, collaborations with medical anthropology have grown in the past 20 years.

It would be totally unrealistic to put forward an elimination of travelling models, which, whatever their limits, also have their utility and effectiveness. Work must be done so that travelling models are more context-sensitive and take better account of the constraints, resources, aspirations, norms and strategies of local or regional actors. However, is it not possible to allocate a little more space, time, attention and money, in the world of public health in general and maternal health in particular, for alternative (qualitative) research focused on pragmatic contexts, and for experiments ‘from the inside’ considering the daily reality of social norms and practical norms? Is this too much to ask?

## Abbreviations

ANC: Antenatal care
FANC: Focused antenatal care
GFATM: Global Fund against Aids, Tuberculosis and Malaria
IDRC: International Development Research Centre
LASDEL: Laboratoire d’études et Recherches sur les Dynamiques Sociales et le Développement Local (Social dynamics and local development research and study laboratory)
LMICs: Low- and middle-income countries
NGO: Non-governmental organisation
PBF: Performance-based financing
PMTCT: Prevention of mother to child transmission of HIV
RBF: Result-based financing
RCT: Randomised control trials
UNICEF: United Nations International Children's Emergency Fund
